# Efficiency of HIV-1 PR-RT genotyping is not impacted by co-infection with HCV

**DOI:** 10.1186/1758-2652-13-S4-P212

**Published:** 2010-11-08

**Authors:** ACA Deloof, H De Wolf, Y Verlinden, J Villacian, T Pattery

**Affiliations:** 1 Vicro bvba, Turhnoutseweg 30, Beerse, Belgium

## Background

Assays that are used for the diagnosis and management of HIV-1 infection are subject to assay interference(s) and co-infection with HCV may interfere with HIV-1 genotyping and/or phenotyping. Since a third of HIV-1 infected patients are frequently HCV co-infected, there is an increasing need to assess the effect of HCV co-infection on the accuracy of the HIV-1 protease (PR) and reverse transcriptase (RT) genotyping.

## Purpose of the study

As a result, the efficiency and accuracy of Virco's HIV-1 PR-RT assay performance [1] was tested on a panel of HIV/HCV co-infected, clinical trial samples.

## Materials and methods

A panel of confirmed 60 HIV/HCV-positive (Hep C antibody +), plasma samples (screening visit), that were collected as part of a HIV-1 clinical trial was PR-RT genotyped (virco®TYPE HIV 1; 1497 bp encoding 1-99 amino acids of PR and 1-400 amino acids of RT), phenotyped (Antivirogram®) and the Clade was determined. In addition, HCV subtyping was performed using NS5B sequence-based subtyping [2] along with NS3/4A genotyping [3] in all of the tested samples. All the 60 samples tested had externally determined plasma HIV viral loads that were >1000 copies/mL.

## Results

For the 60 HIV/HCV + samples tested, the HIV PR-RT target genes were successfully (100%) genotyped, phenotyped and were confirmed to be Clade B. Subtyping (329 bp conserved fragment within NS5B) and genotyping (NS3/4A gene) of the HCV target genes was successful for 49/60 samples (81.6%) and 33 samples were identified as Genotype 1a, 10 samples as 1b, 5 samples as 2b and 1 as genotype 4a.

## Conclusions

We demonstrated that there is no assay interference for HIV-1 genotyping/phenotyping in the presence of an active HCV co-infection. The HIV-1 PR-RT primers used within our certified, high-throughput laboratory is highly sensitive, specific, accurate and reliable to detect HIV-1 clade, genotype and phenotype in HIV/HCV co-infected samples.
